# Digital Sequences and a Time Reversal-Based Impact Region Imaging and Localization Method

**DOI:** 10.3390/s131013356

**Published:** 2013-10-01

**Authors:** Lei Qiu, Shenfang Yuan, Hanfei Mei, Weifeng Qian

**Affiliations:** The State Key Lab of Mechanics and Control of Mechanical Structures, Nanjing University of Aeronautics and Astronautics, 29# Yu Dao Street, Nanjing 210016, China; E-Mails: ysf@nuaa.edu.cn (S.Y.); meihanfei@nuaa.edu.cn (H.M.); kimnuaa@126.com (W.Q.)

**Keywords:** composite structures, impact monitoring, time reversal, digital sequence

## Abstract

To reduce time and cost of damage inspection, on-line impact monitoring of aircraft composite structures is needed. A digital monitor based on an array of piezoelectric transducers (PZTs) is developed to record the impact region of impacts on-line. It is small in size, lightweight and has low power consumption, but there are two problems with the impact alarm region localization method of the digital monitor at the current stage. The first one is that the accuracy rate of the impact alarm region localization is low, especially on complex composite structures. The second problem is that the area of impact alarm region is large when a large scale structure is monitored and the number of PZTs is limited which increases the time and cost of damage inspections. To solve the two problems, an impact alarm region imaging and localization method based on digital sequences and time reversal is proposed. In this method, the frequency band of impact response signals is estimated based on the digital sequences first. Then, characteristic signals of impact response signals are constructed by sinusoidal modulation signals. Finally, the phase synthesis time reversal impact imaging method is adopted to obtain the impact region image. Depending on the image, an error ellipse is generated to give out the final impact alarm region. A validation experiment is implemented on a complex composite wing box of a real aircraft. The validation results show that the accuracy rate of impact alarm region localization is approximately 100%. The area of impact alarm region can be reduced and the number of PZTs needed to cover the same impact monitoring region is reduced by more than a half.

## 1. Introduction

In recent years, large scale composite structures have been widely applied in large aircrafts [1–3]. On-line impact monitoring of these structures on-board and off-board during their whole service lifetime is very important [2–7]. Damage inspection systems such as X-ray systems and ultrasonic C-scan systems are powerful for inspecting small inner impact damages of aircraft composite structures. But they are limited to point-by-point measurements and their use for large scale composite structures is time-consuming. It is inconvenient to apply them to the on-line monitoring under the service environment conditions of composite structures because they are heavy, with high power consumption and high cost.

For realizing health monitoring of structures on-line, guided wave (GW)-based structural health monitoring (SHM) technology is widely studied. The impact monitoring methods and systems are important parts of GW-based SHM. Many impact localization methods have been studied in recent years, such as time differences and geometry methods [8–14], artificial intelligent methods [15–17], mechanical model methods [18,19], directional sensors array methods [20,21] and impact imaging methods [22–29] *etc.* Most of these methods aim to realize impact localization accurately, but all of them need to acquire beforehand the impact response signal output from piezoelectric transducers (PZTs) or acoustic emission sensors. Thus, the hardware architecture of impact monitoring systems should include the following components: signal amplifier circuit, analog to digital (AD) converter and computer systems for supporting the complicated impact localization methods. The following two components should be part of the software architecture: a processing program for complicated signals and an impact localization program. Aiming at engineering applications and high precision impact localization, the Acellent Corporation has developed a series of integrated GW-based SHM systems to monitor damage and impact events [30]. Qiu and Yuan *et al.* developed a GW- and PZTs-based integrated multi-channel scanning system called PXI-ISS to realize both impact and damage monitoring [31,32]. However, since conventional PZTs-based impact monitoring systems are developed to fulfill a high precision impact or damage localization of composite structures, the weight and size of these systems still limits their applications in on-line service. That is why the reported applications of these systems were mainly concentrated on the lab or the ground applications [32–34]. Metis Design Corporation has developed a series of small MD7 digital SHM systems for impact and damage monitoring [35]. Perelli *et al* developed an embedded SHM system with low power consumption to monitor impact events on-line [36]. On-line impact monitoring requires that the monitoring system be low in weight, small in size, have low power consumption and able to access large sparse sensor arrays to cover a large monitoring region. None of the systems mentioned above can meet these requirements at the current stage.

A new concept was put forward by Yuan *et al.* [37,38] to achieve fast and low cost damage inspection of aircraft composite structures. They proposed that if the impact region instead of the accurate impact position can be monitored, damage inspection can be applied on schedule to impact regions only, according to the impact region monitoring reports. The cost and time of damage inspections can then be greatly reduced. Based on the concept, a digital monitor based on PZTs array was developed to record the history of the impact events and give a localized impact alarm region for further damage inspection. In the digital monitor, all the complex analog circuits of conventional impact monitoring systems are replaced by a simple comparators array to turn the output of the PZTs directly into digital sequences. Field programmable gates array (FPGA) is adopted as the processing core to acquire the digital sequences and record the impact events.

Though the digital monitor has all the advantages of small size, light weight and low power consumption, the impact alarm region localization method implemented in the digital monitor needs to be improved according to the two main problems found in its practical engineering application in the recent years. The impact alarm region localization method and the two problems are discussed in the following sections.

**Figure 1.**
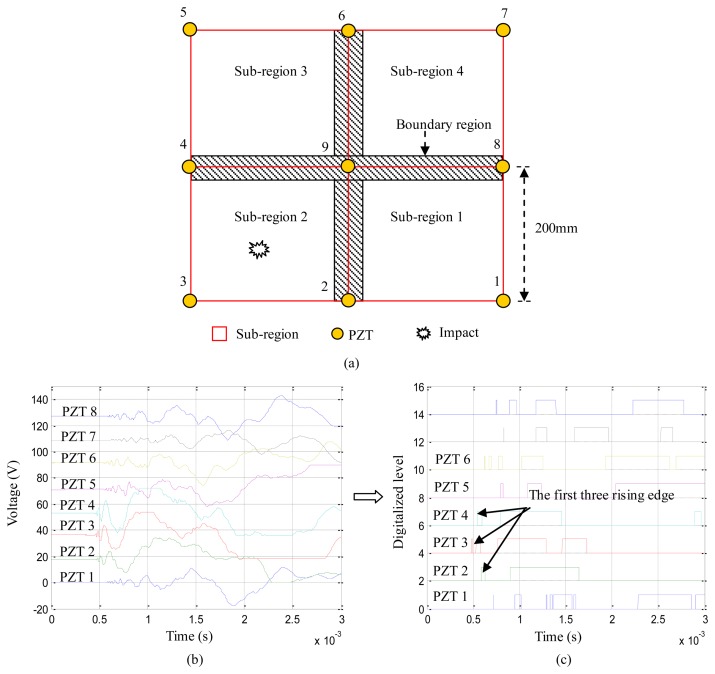
Sub-region dividing based impact alarm region localization method (**a**) PZTs placement on a monitored structure; (**b**) Waterfall plot of impact response signal of PZT1 to PZT8 acquired by Oscilloscope; (**c**) Waterfall plot of digital sequences of PZT1 to PZT8 acquired by digital monitor.

As shown in Figure 1a, nine PZTs are placed on a structure. The impact monitoring region covered by the nine PZTs is divided into four square sub-regions surrounded by every four adjacent PZTs. The area of each sub-region is 200 mm × 200 mm. When an impact occurs in the sub-region 2 surrounded by PZT2, PZT3, PZT4 and PZT9, the impact response signals of PZT1 to PZT8 are acquired and the corresponding digital sequences are acquired by the digital monitor at the same time, just as shown in Figure 1b,c. According to the time-of-flight of the first rising edge appeared in each digital sequence, the first three PZTs (PZT2, PZT3, PZT4) can be recognized. Thus, the impact alarm region can be localized to the sub-region 2. The area of the impact alarm region is equal to the area of the sub-region. This method can be called as a sub-region dividing-based impact alarm region localization method. This method can be easily implemented in the FPGA of the digital monitor because it is very simple, but there are two main problems:

(1) As shown in Figure 1a, there is a boundary region between two adjacent impact alarm regions. When an impact occurs in or near the boundary region, the accuracy rate of impact alarm region localization is low. The width of the boundary region depends on the degree of complexity of the monitored structure. The width of the boundary region is small on a simple plate-like composite structure. But in a real aircraft structure there are many stiffeners, ribs, booms and beams, which are connected to composite plates. The thickness of the composite plates is variable. The area of the boundary region can be larger and even equal to the sub-region.
(2) The area of the impact alarm region shown in Figure 1a is 200 mm × 200 mm. It says that the impact can be localized in the region of 200 mm × 200 mm and the damage inspection only need to be applied in this small area. This result can be acceptable in engineering applications when using the digital monitor, but if the area of impact monitoring region is 2 m × 2 m, more than 120 PZTs should be placed on the structure and at least five digital monitors should be used (24 PZTs can be supported by a single digital monitor). The additional weight of the whole impact monitoring system to monitor only such a small area comparing to large scale structures is not acceptable. Thus, the distance between PZTs must be increased. Taking the distance of 600 mm for example, the structural area of 3 m × 1.8 m can be covered by 24 PZTs and one digital monitor, but then the area of the impact alarm region is enlarged to 600 mm × 600 mm. It means that the time and cost of damage inspection increases nine times.

Basically, the two problems are introduced by the definition of the impact alarm region and the division into sub-regions. Thus, the impact alarm region localization method should be changed and improved.

In recent years, several researchers have reported impact imaging methods [22–29] using PZT- or acoustic sensor-based impact localization taking advantage of the time reversal method [39]. Compared with the time differences and geometry-based impact location methods, impact imaging methods have shown promising advantages by giving a focused image of the structural impact and of higher fault tolerance to velocity errors and signal noises, but most the of impact imaging methods need to acquire the transfer functions of impact signals propagating on the structure beforehand. In engineering applications when a PZT array is placed on an in-service aircraft structure, man-made impacts cannot be allowed to be applied on the structure to acquire any *a priori* knowledge about the transfer functions. To monitor complex composite structures, the transfer functions are hard to obtain by theoretical modeling methods or finite element methods. Even if the transfer functions are acquired, they cannot be used to handle the digital sequences of the digital monitor. Considering these problems, a phase synthesis time reversal impact imaging method which does not rely on transfer functions can be adopted [25], but to acquire accurate impact localization results, narrow frequency band signals extracted from the impact response signals are needed to accomplish the time reversal focusing. The digital monitor cannot acquire the impact response signals from the PZTs because the analog circuits such as amplifier and AD converter are replaced by a simple array of comparators. Thus, the phase synthesis time reversal impact imaging method cannot be used directly by the digital monitor.

To address the two problems mentioned above and take advantage of the time reversal based impact imaging method, an impact alarm region imaging and localization method based on digital sequences and time reversal is proposed. It can increase the localization accuracy rate and reduce the area of the impact alarm region.

Considering the following two aspects, the phase synthesis time reversal impact imaging method is adopted based on some simplifications: (1) only the impact alarm region needs to be localized but not the accurate impact position because the digital monitor can only acquire digital sequences but not impact response signals; (2) for further study, a simple, stable and low power consumption method is more suitable because the whole method needs to be implemented in the FPGA to fulfill the on-line and on-board impact monitoring needs.

In the impact alarm region imaging and localization method, the frequency band of the impact response signals is first estimated based on digital sequences. Then, the characteristic impact response signals are constructed by sinusoidal modulation signals. Finally, the impact imaging method is adopted to obtain the impact region image. Depending on the image, an error ellipse is generated to delineate the final impact alarm region. This paper is organized as follows:

(1) In Section 2, the hardware architecture of the digital monitor developed by the authors in the recent past is discussed briefly.
(2) In Section 3, the impact alarm region imaging and localization method is proposed and the details of the method are studied including the following contexts: (1) the frequency band of the impact response signals is discussed and a frequency band estimation method based on digital sequences is studied; (2) the frequency band of the sinusoidal modulation signals is analyzed. Combining with the frequency band estimation method, a frequency narrow band characteristic signals constructing method based on sinusoidal modulation signals is studied; (3) the phase synthesis time reversal impact imaging method is discussed briefly and an error ellipse-based impact alarm region estimation method is studied; (4) the process of implementation of the whole method is summarized.
(3) In Section 4, an experiment is implemented on the composite wing of a real aircraft to validate the performance of the method.

## 2. The Hardware Architecture of the Digital Monitor

Based on its application in recent years, the digital monitor has been improved:

(1) A digital sequence software de-noising method is proposed to reduce the high frequency noises [40].
(2) In order to reduce the load noises, a high-pass filter array is added to the hardware of the digital monitor.
(3) The power consumption is reduced and the reliability is improved.

**Figure 2.**
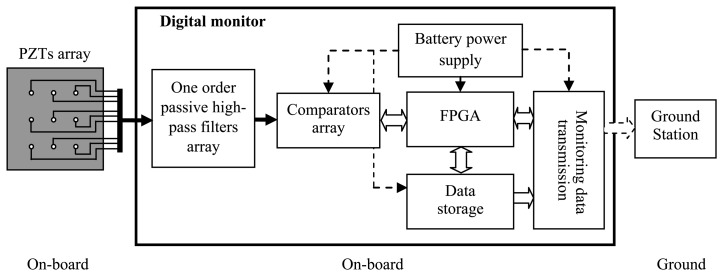
Hardware architecture of the improved digital monitor.

Figure 2 shows the hardware structure of the improved digital monitor which mainly consists of a one order passive high-pass filter array, comparator array, FPGA, data storage, data transmission monitoring and battery power supply. The digital monitor is small size (8 × 6 × 3 cm^3^), lightweight (lighter than 120 g), low power consumption (lower than 80 mW) and can access up to 16 or 24 PZTs.

When an impact occurs on a monitored structure, impact signals will be generated and be obtained by the PZT array. Then, the impact response signals of the PZTs are transmitted to the filter array of the digital monitor through shielded cables. A one order passive high-pass filter array of −3 dB with a cut-off frequency of 8 kHz is designed to reduce the low frequency load noises when the aircraft is in-flight. The comparators array converts the filtered impact response signals to digital sequences which are acquired by the FPGA. The digital sequences and impact alarm region localization results are stored in the data storage system. The digital monitor is fixed on the aircraft and implements impact monitoring on-board and off-board. The monitoring results can be downloaded by a ground station when the aircraft is on the ground.

## 3. Impact Alarm Region Imaging and Localization Method

In this section, a new definition of impact alarm region is discussed first. Then, the impact alarm region imaging and localization method is discussed in detail, including the frequency band estimation method, the characteristic signals constructing method, the phase synthesis time reversal impact imaging method and the error ellipse. Finally, the implementation process of the impact alarm region imaging and localization method is described.

### 3.1. New Definition of Impact Alarm Region

**Figure 3.**
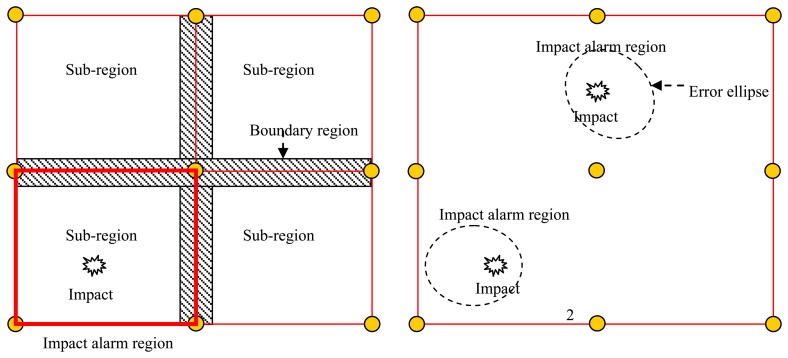
Illustration of new definition of impact alarm region. (**left**) Impact alarm region definition of the digital monitor; (**right**) New definition of impact alarm region.

As mentioned in Section 1, two problems are introduced by the definition of the impact alarm region and the dividing sub-regions. In the impact alarm region localization method, the sub-region is decided and fixed by the placement of the PZT array shown in Figure 3(left). When an impact occurs in one sub-region, the impact alarm is generated and the area of the impact alarm region is equal to the area of the sub-region, but if the position and area of the impact alarm region can be changed automatically according to the impact position, the boundary is eliminated and the impact alarm region area can be reduced. Thus, a new definition of impact alarm region is proposed and shown in Figure 3(right). When an impact occurs, an error ellipse region can be calculated based on the digital sequences. If the real impact position is covered by the region surrounded by the error ellipse, the error ellipse region can be considered to be the impact alarm region, but if the real impact position is not covered by the region surrounded by the error ellipse, the error ellipse region is considered to be a false impact alarm region.

If the impact can be given out as an image of the impact monitoring region, the error ellipse can be acquired easily and intuitively. A time reversal-based impact imaging method can be adopted to realize this purpose. Among the impact imaging methods, the phase synthesis time reversal impact imaging method can fulfill the time reversal focusing in software and does not rely on transfer functions of the impact signal propagating on the structure. Thus, this paper adopts the phase synthesis time reversal imaging method to obtain the impact image approximately, but narrow band frequency signals of the impact response signals are required in this method at the current stage and cannot be used directly. To address this problem, a digital sequences-based frequency band estimation method and characteristic signals construction method are proposed, which are discussed in Sections 3.2 and 3.3, respectively.

### 3.2. Digital Sequences Based Frequency Band Estimation Method

In engineering applications when a PZT array is placed on an in-service aircraft structure, man-made impacts cannot be allowed to be applied on the structure to acquire any *a priori* knowledge about impact response signals and different impacts have different frequency bands. The frequency band of impact response signals needs to be estimated depending on the digital sequences acquired by the digital monitor in the on-line impact monitoring application. Thus, a digital sequences-based frequency band estimation method should be studied first.

#### 3.2.1. Impact Response Signals, Digital Sequences and Frequency of Rising Edge

**Figure 4.**
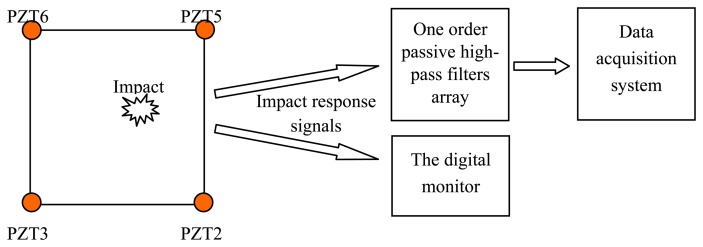
Experimental setup of impact response signal acquiring.

**Figure 5.**
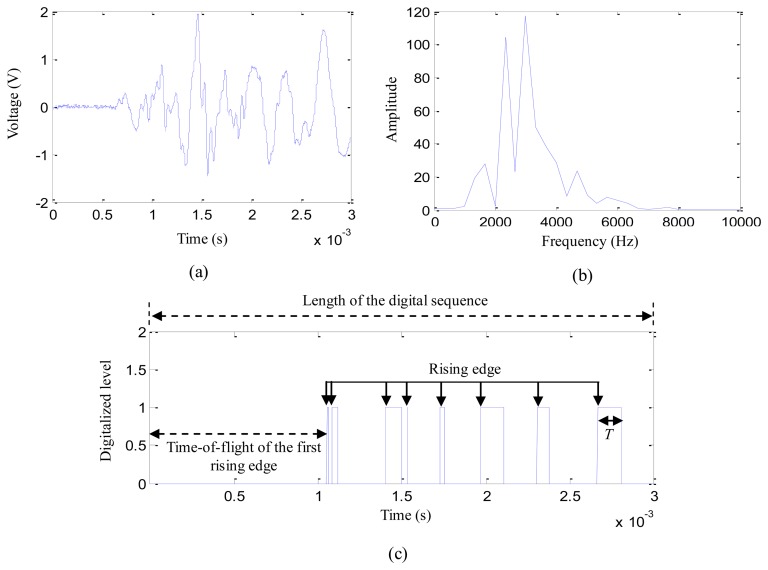
Typical impact response signal of PZT and the digital sequence. (**a**) Impact response signal of PZT3; (**b**) Frequency spectrum; (**c**) Digital sequence of impact response signal of PZT 3.

A simple impact experiment is implemented on the structure which is discussed in Section 4. The experimental setup is shown in Figure 4. Nine impacts of 15 J impact energy are applied at random positions in the region surrounded by PZT2, PZT3, PZT5 and PZT6. The impact response signals output by the four PZTs are input to one order passive high-pass filter array which is same as the filter array used in the digital monitor. The final outputs are acquired by a data acquisition system. At the same time, the outputs of the PZTs are connected to the digital monitor. Figure 5a,b give out a typical impact response signal of PZT3 and the corresponding frequency spectrum, respectively. It indicates that the main energy of the impact response signal is concentrated in the frequency range of 1.5 kHz to 6 kHz before it is digitalized. A comparator with a comparison voltage of 0.5 V is applied in the software to the impact response signal. It is digitalized as shown in Figure 5c.

**Figure 6.**
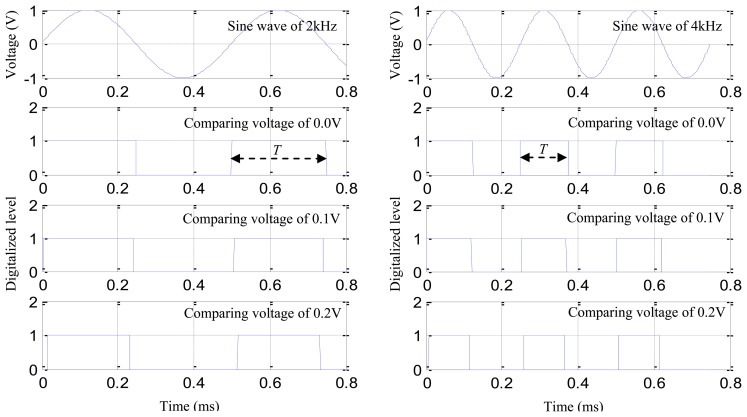
Sine waves and the digital sequences. (**left**) Sine wave of 2 kHz and the digitalized sequences of different comparing voltage; (**right**) Sine wave of 4 kHz and the digitalized sequences of different comparing voltage.

In the digital sequence, the duration time of a single rising edge is denoted as *T*. The length of *T* depends on the frequency of signal component which introduces the corresponding rising edge in the impact response signal. The reason is explained as follows: two sine waves of 2 kHz and 4 kHz are constructed as shown in Figure 6. The sampling rate is 1 MHz. The comparison voltages of 0 V, 0.1 V and 0.2 V are adopted to digitalize the sine waves, respectively. Comparing the digitalized sequences shown in Figure 6(left) with the digitalized sequences shown in Figure 6(right), the length of *T* becomes shorter when the frequency of the sine wave increases. By comparing the digital sequences of different comparison voltages in Figure 6(left), it can be noted that the length of *T* becomes shorter accompanying with the increasing comparing voltage. Figure 6(right) shows the same.

If the *T* is adopted to calculate the frequency of the sine waves, the results can be obtained shown in Table 1 by using the following equation:

(1) $$F_{SD}=\frac{1}{2T}$$

where, *F_SD_* is defined as the frequency of single rising edge.

The results of Table 1 show that the frequency of rising edge is equal to the real frequency of the sine wave when the comparing voltage is 0.0 V. The frequency of the rising edge becomes higher than the real frequency of the sine wave when the comparing voltage is increased.

According to the superposition principle, the impact response signal consists of many sine waves of different frequency. Thus, based on the discussion, it can say that the frequency of a single rising edge in a digital sequence can be used to indicate approximately the frequency components of the impact response signal.

#### 3.2.2. Frequency Band Estimation of Impact Response Signals

As shown in Table 1, the minimum calculation error can be obtained when the comparing voltage is 0 V. But this comparing voltage cannot be adopted in real applications because of signal noises. Thus, the *F_SD_* is always higher than the frequency of impact response signal. To reduce the frequency band estimation error, the minimum frequency of single digital sequence *F_i-SD_*_min_ is defined as follows:

(2) $$F_{i-SD\min}=\min\{F_{SDj},j=1,\ldots ,m\}i=1,\ldots ,n$$

where *n* denotes the number of the digital sequences and *i* denotes the digital sequence number. *m* denotes the number of rising edges of the digital sequence and *i*, *j* denotes the rising edge number.

**Figure 7.**
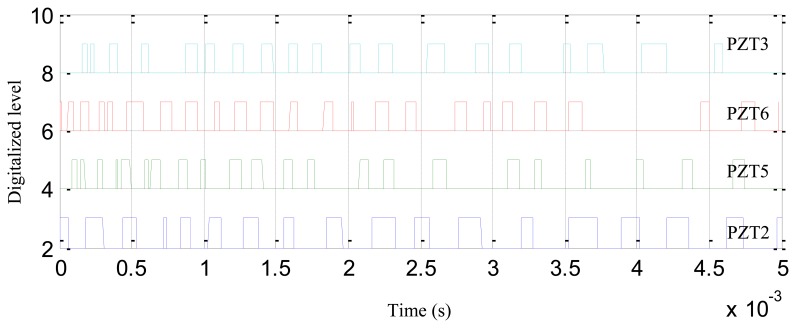
Typical digital sequences acquired by the digital monitor.

Figure 7 shows the waterfall plot of the digital sequences acquired by the digital monitor in the experiment discussed in Section 3.2.1. The sampling rate and sampling dots of the digital sequences are set to be 1 MHz and 5,000 in the digital monitor, respectively.

By using Equations (1) and (2), the frequency calculation results of the digital sequences can be obtained and are shown in Table 2. The results of the minimum frequency in the table are in the range of the frequency band of the impact response signal obtained in Section 3.2.1. Thus, the final frequency band estimation result *F_band_* and the central frequency *F_cen_* of digital sequences are defined as follows: according to Table 2, the frequency band estimation result is [2,392 Hz, 5,434 Hz]:

(3) $$\left\{ \begin{array}{l} F_{L}=\min\{F_{i-SD\min},i=1,\ldots ,n\}\\ F_{H}=\max\{F_{i-SD\min},i=1,\ldots ,n\}\\ F_{band}=[F_{L},F_{H}],F_{cen}=(F_{H}+F_{L})/2 \end{array}\right.$$

### 3.3. Characteristic Signal Construction Method

As mentioned in Section 1, the narrow frequency band signals are needed in the phase synthesis time reversal process. Active Lamb wave based damage monitoring technology [41] usually adopts sinusoidal modulation signals to excite Lamb wave signals of the frequency narrow band on structures and the frequency band of Lamb wave response signals output by the PZTs is in the range of the frequency band of the excitation signals. Thus, this paper adopts sinusoidal modulation signals to construct the characteristic signals of the impact response signals based on digital sequences.

#### 3.3.1. Sinusoidal Modulation Signal and the Frequency Band Analysis

The expression of sinusoidal modulation signal is represented as the following equation:

(4) $$u(t)=(1-\cos\frac{2\pi f_{c}t}{N})\sin(2\pi f_{c}t)$$

where *N* is the number of cycles of sine wave. *f_c_* is the central frequency of the sine wave. The frequency response of sinusoidal modulation signal can be obtained by applying the Fourier transform to Equation (4):

(5) $$U(\omega )=\int_{-\infty }^{+\infty }u(t)e^{-j\omega t}dt=\int_{-\infty }^{+\infty }\left(1-\cos\frac{\omega_{c}t}{N}\right)\sin(\omega_{c}t)e^{-j\omega t}dt$$

where *ω_c_* =2*πf_c_*. The Fourier transform of exponential function can be represented as follows:

(6) $$\int_{-\infty }^{+\infty }e^{j\omega_{c}t}e^{-j\omega t}dt=2\pi \delta (\omega -\omega_{c})$$

where:

$$\delta (\omega )=\{\begin{array}{cc} 1 & \omega =0\\ 0 & \omega \neq 0 \end{array}$$

Thus, Equation (5) can be changed to Equation (7):

(7) $$U(\omega )=j\pi \left\{\delta \left(\omega -\frac{N+1}{N}\omega_{c}\right)-\delta \left(\omega +\frac{N-1}{N}\omega_{c}\right)\right\}+j\pi \left\{\delta \left(\omega -\frac{N-1}{N}\omega_{c}\right)-\delta \left(\omega +\frac{N+1}{N}\omega_{c}\right)\right\}+j\pi \left\{\delta (\omega -\omega_{c})+\delta (\omega +\omega_{c})\right\}$$

By considering the positive frequency components, Equation (7) can be changed to Equation (8):

(8) $$U(\omega )=\frac{j\pi \delta \left(\omega -\frac{N+1}{N}\omega_{c}\right)-j\pi \delta \left(\omega +\frac{N-1}{N}\omega_{c}\right)}{2}+j\pi \delta (\omega -\omega_{c})$$

Thus, the normalized amplitude of the frequency response can be obtained as shown in Equation (9). It indicates that frequency band of the sinusoidal modulation signal is (2/*N*)*ω_c_*:

(9) $$|U(\omega )|=\{\begin{array}{ll} 1 & \omega =\omega_{c}\\ \frac{1}{4} & \omega =\frac{N+1}{N}\omega_{c}\\ \frac{1}{4} & \omega =\frac{N+1}{N}\omega_{c} \end{array}$$

#### 3.3.2. Characteristic Signals of Impact Response Signals

**Figure 8.**
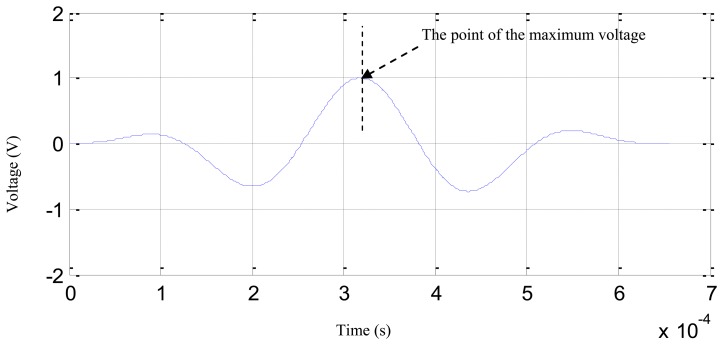
Constructed normalized sinusoidal modulation signal of frequency band of [2,392 Hz, 5,434 Hz].

**Figure 9.**
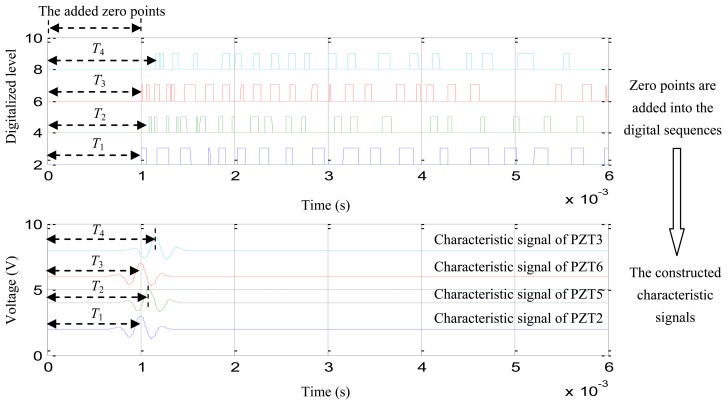
The demonstration of the characteristic signals construction.

Combining Equations (3) and (9), the central frequency, the number of cycles and the time of the sinusoidal modulation signal can be calculated by Equation (10). Thus, the sinusoidal modulation signal can be constructed according to the frequency band estimation result:

(10) $$\left\{ \begin{array}{l} f_{c}=\frac{F_{H}+F_{L}}{2}\\ N=\frac{F_{H}+F_{L}}{F_{H}-F_{L}}\\ t_{start}=\frac{N}{f},t_{end}=\frac{N}{f},t_{\mathrm{int}erval}=\frac{1}{f_{s}} \end{array}\right.$$

where, the time denoted as *t* in Equation (4) is *t_start_* from to *t_end_* and *t_interval_* is the time interval. Once the frequency band estimation result is obtained, the characteristic signals can be constructed by the following steps:

(1) Based on the frequency band estimation result of [2,392 Hz, 5,434 Hz] and the 1 MHz sampling rate of the digital monitor, the parameters of *f_c_*, *N*, *t_start_*, *t_end_* and *t_interval_* are obtained according to Equation (10): *f_c_* = 3,913 Hz, *N* = 2.57, *t_start_* = 2.57 × 10^−6^ s, *t_end_* = 6.57 × 10^−4^ s and *t_interval_* = 1 × 10^−6^.
(2) Substituting the parameters into Equation (4), the normalized sinusoidal modulation signal is constructed as shown in Figure 8.
(3) Zero points are added before each digital sequence of the digital sequences shown in Figure 7 and the time-of-flight of the first rising edge of the zero points plus the added digital sequences is calculated, just as shown in Figure 9(top).
(4) The point of the maximum voltage of the normalized sinusoidal modulation signal is adjusted to be equal to the time-of-flight of the first rising edge. Finally, the characteristic signals are constructed as shown in Figure 9(bottom).

### 3.4. The Phase Synthesis Time Reversal Impact Imaging Method and the Error Ellipse

The phase synthesis time reversal impact imaging method [25] fulfills the time reversal process by applying phase delay factors to the frequency narrow band signals of impact response signals directly in software. In this method, the complex Shannon wavelet transform is used to extract frequency narrow band signals from impact response signals and measure the group velocity of the signals. The simplified phase synthesis expression is represented by Equation (11):

(11) $$\left|V(t)\right|\approx \sum_{i=1}^{n}\text{Envelope}\left(e_{i}\left(\tau -t+\frac{r_{i}}{C_{g}}\right)\right)$$

where *V*(*t*) is the phase synthesis signal. *e_i_*(*t*) is the frequency narrow band signal extracted from the impact response signals of the *i*th PZT. *C_g_* is the group velocity of the frequency narrow band signals. *r_i_* is the propagation distance. *Envelope* denotes the positive envelope of the frequency narrow band signal. *n* is the number of PZTs.

In this paper, the *e_i_*(*t*) is used instead of the characteristic signals. Equation (11) can be changed to Equation (12), which indicates that the envelope and the group velocity are needed:

(12) $$\left|V(t)\right|\approx \sum_{i=1}^{n}\text{Envelope}\left(CS_{i}\left(\tau -t+\frac{r_{i}}{C_{g}}\right)\right)$$

where *CS_i_*(*t*) is the characteristic signal of the PZT *i*.

There are many methods that can be adopted to acquire the envelope of a signal. Thus, this paper does not discuss this aspect in any detail. To obtain the group velocity, the active Lamb wave and complex Shannon wavelet transform based velocity measuring method is adopted [25].

**Figure 10.**
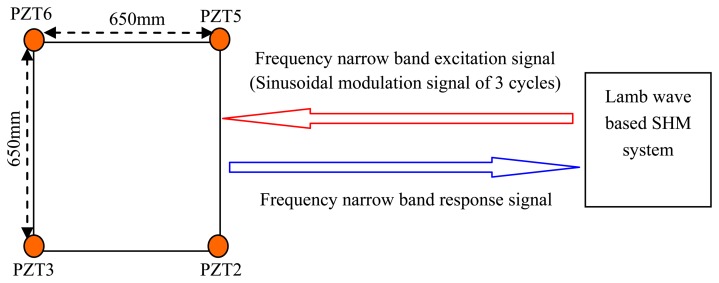
Experimental setup of group velocity measuring.

A simple velocity measuring experiment is implemented on the structure which is discussed in Section 4. The experimental setup is shown in Figure 10. The GW-based SHM system adopted in the experiment is called a multi-channel PZT array scanning system [32]. The frequency narrow band excitation signal is output to PZT2 and the frequency narrow band response signals of the other three PZTs are acquired by the system. Though the frequency band estimation results cannot be obtained beforehand, the group velocity is only dependent on the central frequency of the signal and the thickness of the structure. In anisotropic structures the group velocities also depend on the direction of propagation along the structure, but considering that the method is region imaging not precise imaging, the average group velocity is adopted.

**Figure 11.**
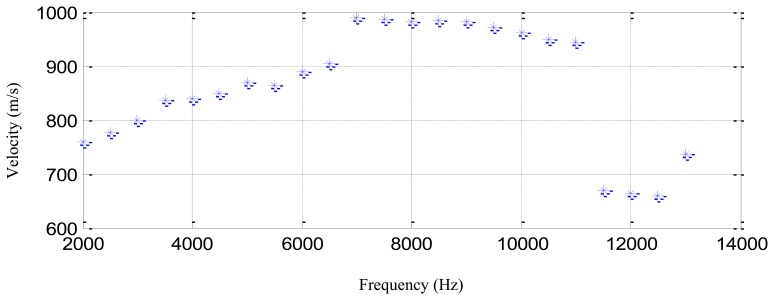
The averaged group velocity measuring results of different central frequencies.

The sinusoidal modulation signal of three cycles is adopted to be the excitation waveform and the central frequency is changed from 2 kHz to 13 kHz of 0.5 kHz interval. The thickness of the structure is 10 mm to 11 mm. In one central frequency, three group velocity measuring results can be obtained from the actuator-sensor channels of PZT2-PZT5, PZT2-PZT6 and PZT2-PZT3, respectively. The averaged group velocity is considered to be the final measuring result. Figure 11 shows the averaged group velocity measuring results of different central frequencies. It indicates that the group velocities are in the range of 650 m/s to 1,000 m/s. The average group velocity of 865 m/s is adopted.

**Figure 12.**
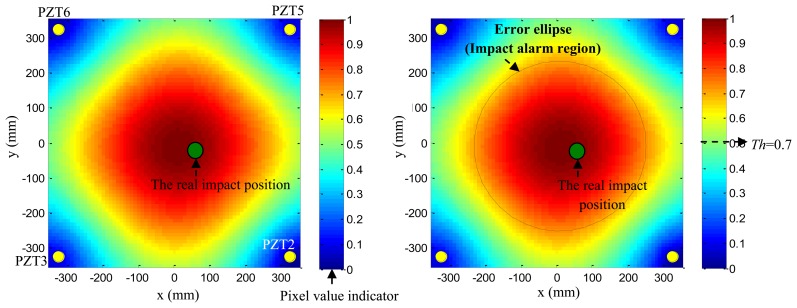
Illustration of impact imaging result and the impact alarm region. (**left**) Impact alarm region imaging result; (**right**) Error ellipse based impact alarm region estimation.

Based on the phase synthesis time reversal impact imaging method, the impact alarm region image can be obtained as shown in Figure 12(left). In this paper, the method is performed on a personal computer, thus, the image resolution is set to be 1 mm × 1mm, which is relatively high, but the higher the resolution is, the lower the computation speed is. In further work, the imaging method will be performed in the FPGA, and the resolution will be decreased depending on the computation ability of the FPGA and the time. The relationship between the image resolution and the final impact alarm region localization result will be discussed in further work. The points of pixel values of the image represent the amplitude of the phase synthesis signals. Supposing the point of the largest pixel value of the image to be (*x*_c_,*y*_c_) and a pixel value threshold to be *Th* (*Th* is set to be 0.7 in this example), all the points (*x_k_*,*y_k_*) whose pixel values are higher than *Th* can be obtained. The number of the points is denoted as *k*. The half-length of the error ellipse axis in the X direction and Y direction can be calculated by Equation (13):

(13) $$A_{x}=\sqrt{\frac{{\left(x_{k}-x_{c}\right)}^{2}}{k}},A_{x}=\sqrt{\frac{{\left(y_{k}-y_{c}\right)}^{2}}{k}}$$

As shown in Figure 12(right), the error ellipse can be generated based on the parameters of (*x*_c_,*y*_c_), *A_x_* and *A_y_*. The region surrounded by the error ellipse is considered to be the final impact alarm region. If the real impact position is covered in the region of the error ellipse, the impact alarm region localization is correct. The area of region surrounded by the error ellipse can be calculated by S =*πA_x_A_y_*. It indicates that the area depends on the *Th*. If the *Th* is low, the accuracy rate of impact alarm region localization is high, but the area is large. If the *Th* is high, the area becomes smaller and the accuracy rate decreases.

Though, the phase synthesis time reversal impact imaging method can be applied to the digital sequences based on the studies, it makes many approximations, but considering that only the impact alarm region needs to be localized, not the accurate impact position, the approximations can be accepted.

### 3.5. The Implementation Process of the Impact Alarm Region Imaging Method

**Figure 13.**
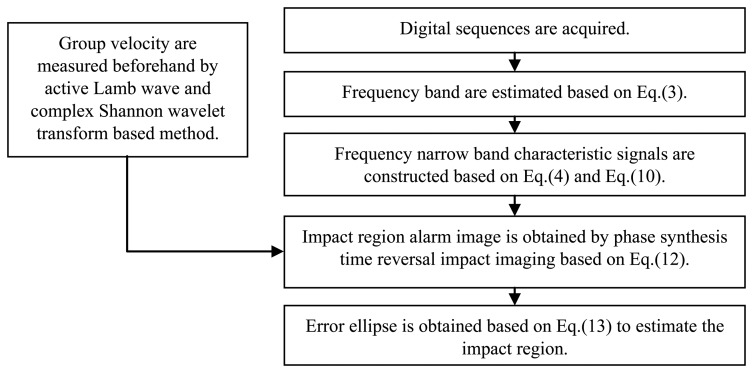
Implementation process of the impact alarm region imaging method.

Based on the discussions and studies in Sections 3.1 to 3.4, the implementation process of the impact alarm region imaging method is summarized as shown in Figure 13. This method will be implemented in FPGA in further study. In this paper, it is implemented by MATLAB on a computer to validate it.

## 4. Validation Experiment on a Complex Composite Wing Box

### 4.1. Validation Experimental System and the Experimental Setup

**Figure 14.**
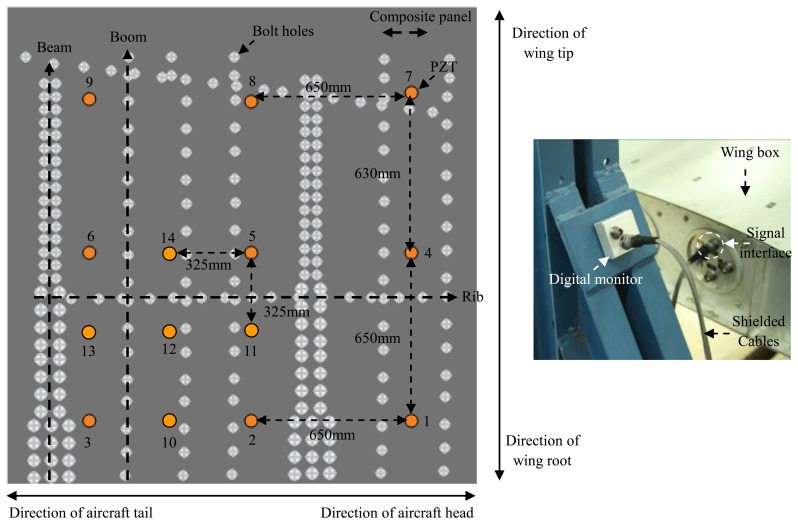
Illustration of the validation experimental system.

The validation experimental system shown in Figure 14 consists a complex composite wing box, the digital monitor and the PZT array. The composite wing box contains two beams, five booms, two ribs and composite panels, which are all made of carbon fiber-reinforced composite materials.

Fourteen PZTs are placed on the inner surface of the composite panel. PZT1, PZT4, PZT7, PZT8, PZT9 and PZT3 make up the impact monitoring region 1. PZT2, PZT5, PZT6, PZT3 and PZT11 to PZT14 constitute the impact monitoring region 2. The areas of region 1 and region 2 are 1,300 mm × 1,280 mm and 650 mm × 650 mm, respectively. The thinnest and the thickest of the thicknesses of the composite panel are 10 mm and 11 mm, respectively. All the PZTs are connected to the digital monitor through shielded cables. The sampling rate and sampling length of the digital sequences in the digital monitor is set to be 1 MHz and 5,000 points, respectively. The comparison voltage of the comparators array is set to be 0.5 V.

**Figure 15.**
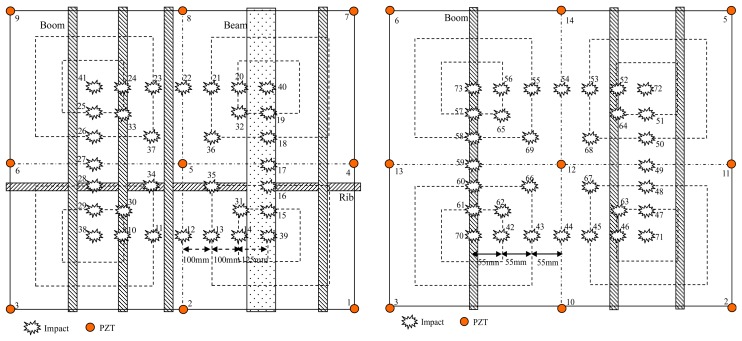
Illustration of impact numbers and positions in the validation experiment. (**left**) Impacts applied in impact monitoring region 1. (**right**) Impacts applied in impact monitoring region 2.

Seventy three impacts of 15 J impact energy are applied in the validation experiment. The impacts 1 to 9 are used in the experiment discussed in Section 3.2.1. The other 64 impacts numbered 10 to 73 are shown in Figure 15. When all the impacts have been applied, a laptop computer is used to download the digital sequences stored in the digital monitor and perform the impact alarm region imaging and localization.

### 4.2. Validation Results and Discussion

According to the sub-region division-based impact alarm region localization method, the two impact monitoring regions can be divided into four sub-regions by nine PZTs. The area of the impact alarm region is 650 mm × 630 mm (25% of the impact monitoring area) and 325 mm × 325 mm (25% of the impact monitoring area), respectively. The impact alarm region localization accuracy rates of the two monitoring regions obtained by using this method are 71% and 92%, respectively. The accuracy rate of impact monitoring region 1 is low and nine PZTs are needed to cover the 1,300 mm × 1,280 mm area of the impact monitoring region. Though the accuracy rate of impact monitoring region 2 is relatively high, nine PZTs are needed to cover such a small impact monitoring region area. The results also indicate that the accuracy rate decreases when the area of impact monitoring region becomes larger because of the anisotropic propagation of impact signals.

**Figure 16.**
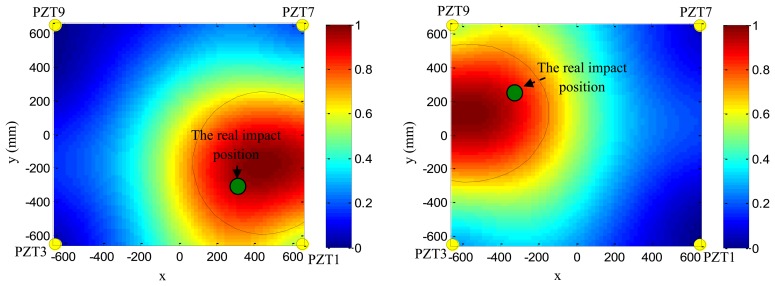
Typical impact alarm region imaging results of impact monitoring region 1: (**left**) imaging result of impact 39; (**right**) imaging result of impact 41.

**Figure 17.**
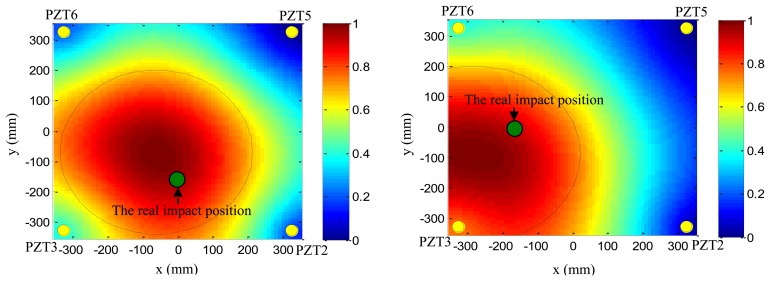
Typical impact alarm region imaging results of impact monitoring region 2: (**left**) imaging result of impact 44; (**right**) imaging result of impact 59.

To validate the impact alarm region imaging and localization method, PZT2, PZT5, PZT6 and PZT3 are adopted to cover the impact monitoring region 1. PZT1, PZT7, PZT9 and PZT3 are adopted to cover the impact monitoring region 2. Figures 16 and 17 show some typical impact alarm region imaging results (*Th* = 0.7). They show that the real impact position can always be covered by the error ellipse. Tables 3 and 4 give the static results of the impact alarm region localization obtained by using the impact alarm region imaging and localization method.

The results show that the accuracy rates of impact alarm region localization of the two monitoring regions under the condition of *Th* = 0.5 are both 100%. The real impact position of impacts 26 and 27 in impact monitoring region 1 are not covered in the region surrounded by the corresponding error ellipses when *Th* =0.7, but in impact monitoring region 2, all impact alarm regions are localized correctly. This result shows that the higher the *Th* is, the lower the localization accuracy rate is. By comparing the results of Tables 3 and 4 when *Th* = 0.7, it also shows that the localization accuracy rate decreases when the area of impact monitoring region becomes larger because of the anisotropic propagation of impact signals. For impact monitoring region 1, the averaged percentages of the area of the impact alarm region to the area of the whole impact monitoring region are 10.6% and 5.0% when *Th* = 0.5 and *Th* = 0.7, respectively. For impact monitoring region 2, the averaged percentages of the area of the impact alarm region to the area of the whole impact monitoring are 21.8% and 12.5%. Comparing the area of impact alarm region of 25% by using the sub-region dividing based impact alarm region localization method and nine PZTs, the area of the impact alarm region is reduced and only four PZTs are needed to cover the impact monitoring region by using the impact alarm region imaging and localization method

## 5. Conclusions

A digital sequences and time reversal-based impact alarm region imaging and localization method are proposed in this paper to improve the impact alarm region localization method implemented in the digital monitor at the current stage. It can increase the accuracy rate of the impact alarm region localization and reduce the area of the impact alarm region.

The digital sequences-based frequency band estimation method is studied. Based on the method, the frequency band of real impact response signals can be estimated approximately only depending on the digital sequences acquired by the digital monitor. A signal construction method used to construct characteristic signals of real impact response signals to fulfill the time reversal focusing and imaging is studied. Finally, the phase synthesis time reversal impact imaging method is adopted to obtain the impact region image. An error ellipse is generated based on images to give the final impact alarm region. A validation experiment is implemented on a complex composite wing box of a real aircraft and it shows that the accuracy rate of the impact alarm region localization is approximately 100%. The area of the impact alarm region can be reduced and the number of PZTs needed to cover the same impact monitoring region is reduced by more than a half.

The size and power consumption of the digital monitor developed by Yuan *et al* is 8 × 6 × 3 cm^3^ and is weighs less than 120 g. It can access up to 16 or 24 PZTs. Based on the proposed impact alarm region imaging method, the total impact monitoring region can be enlarged two times at least and the impact alarm region localization accuracy is increased to approximately 100%. Further work is ongoing to study an impact energy degree estimation method based on digital sequences and implement these methods in the FPGA to implement a more comprehensive on-line impact monitoring system.

## Figures and Tables

**Table 1.** The calculation frequency of sine wave by using the length of a single rising edge.

**Comparing Voltage (V)**	**Frequency of Sine Wave (Hz)**	**Calculate Frequency by Using*T* (Hz)**
0.0	2,000	4,000
0.1	2,127	4,237
0.2	2,304	4,629

**Table 2.** The calculation results of the rising edge frequency of the digital sequences in Figure 7.

**Rising Edge Number**	***F****_SDj_***of the Digital Sequence of PZT2 (Hz)**	***F****_SDj_***of the Digital Sequence of PZT5 (Hz)**	***F****_SDj_***of the Digital Sequence of PZT6 (Hz)**	***F****_SDj_***of the Digital Sequence of PZT3 (Hz)**
*j* = 1	7,936	19,230	27,777	7,042
*j* = 2	4,000	16,129	13,888	19,230
*j* = 3	5,000	16,129	7462	8,928
*j* = 4	20,000	33,333	12,195	11,363
*j* = 5	6,666	7,692	15,151	6,024
*j* = 6	5,882	27,777	4,310	7,812
*j* = 7	4,504	7,246	5,882	7,042
*j* = 8	6,666	9,259	6,172	6,578
*j* = 9	4,310	13,513	14,285	7,692
*j* = 10	3,105	6,756	5,952	8,474
*j* = 11	4,807	7,042	5,319	6,493
*j* = 12	3,164	8,064	8,771	5,319
*j* = 13	6,024	10,869	7,812	4,000
*j* = 14	2,392	7,575	250,000	5,747
*j* = 15	3,937	6,250	71,428	6,410
*j* = 16	3,246	5,434	5,747	9,615
*j* = 17	4,098	6,666	7,042	4,545
*j* = 18	/	10,869	6,250	2,840
*j* = 19	/	11,904	11,627	8,771
*j* = 20	/	9,433	7,246	/
*j* = 21	/	7,142	6,024	/
*j* = 22	/	6,666	5,434	/
*j* = 23	/	/	8,333	/
*j* = 24	/	/	5,494	/
Minimum frequency	*F*_1_*_-Sd_*_min_ =2,392	*F*_2_*_-SD_*_min_ =5,434	*F*_3_*_-SD_*_min_ =4,310	*F*_4_*_-SD_*_min_ =2,840

**Table 3.** Results of impact monitoring region 1 obtained by using impact alarm region imaging method.

**Impact number**	**Central Coordinate of Error Ellipse**		**Half length of the Error Ellipse Axis (*Th* = 0.5)**		**Percentage of the Impact Alarm Area to the Impact Monitoring area (%) (*Th* = 0.5)**	**Half length of the Error Ellipse Axis (*Th* = 0.7)**		**Percentage of the Impact Alarm Area to the Impact Monitoring area (%) (*Th* = 0.7)**
		
	**x** **(mm)**	**y** **(mm)**	**X direction** **(mm)**	**Y direction** **(mm)**		**X direction** **(mm)**	**Y direction** **(mm)**	
10	−310	−270	191	198	7.1	144	134	3.6
11	−170	−110	245	268	12.4	230	250	10.9
12	−170	−650	262	303	15.0	209	220	8.7
13	50	−190	201	218	8.3	138	147	3.8
14	90	−350	349	209	13.8	176	177	5.9
15	590	−110	255	241	11.6	178	155	5.2
16	290	−90	209	216	8.5	162	141	4.3
17	330	70	202	220	8.4	163	142	4.4
18	390	90	206	241	9.4	157	155	4.6
19	430	130	201	230	8.7	148	150	4.2
20	330	310	206	212	8.2	161	164	5.0
21	30	470	238	216	9.7	156	151	4.4
22	−90	290	216	208	8.5	140	161	4.3
23	−190	270	232	214	9.4	154	166	4.8
24	−270	350	219	205	8.5	155	157	4.6
25	−450	190	414	311	24.3	160	220	6.6
**26**	−650	190	271	246	12.6	**179**	**221**	7.5
**27**	−650	50	205	303	11.7	**110**	**200**	4.2
28	−650	170	482	238	21.7	134	256	6.5
29	−410	−170	201	239	9.1	152	152	4.4
30	−390	−170	204	238	9.2	157	153	4.5
31	210	−50	222	209	8.8	152	142	4.1
32	150	170	208	212	8.3	141	143	3.8
33	−330	90	202	221	8.4	163	144	4.4
34	−310	−70	222	242	10.1	174	154	5.1
35	−70	−150	201	208	7.9	137	141	3.6
36	70	110	218	219	9.0	150	150	4.2
37	−230	90	220	216	9.0	156	143	4.2
38	−610	−230	257	233	11.3	178	163	5.5
39	450	−170	209	241	9.5	152	157	4.5
40	490	110	218	249	10.2	152	159	4.6
41	−570	130	240	238	10.8	162	154	4.7
			**Accuracy rate 100%**		**Averaged percentage 10.6%**	**Accuracy rate 94%**		**Averaged percentage 5.0%**

**Table 4.** Results of impact monitoring region 2 obtained by using impact alarm region imaging method.

**Impact number**	**Central Coordinate of Error Ellipse**		**Half Length of the Error Ellipse Axis (*Th*** = **0.5)**		**Percentage of the Impact Alarm area to the Impact Monitoring area (%) (*Th* = 0.5)**	**Half Length of the Error Ellipse Axis (*Th* = 0.7)**		**Percentage of the Impact Alarm Area to the Impact Monitoring area (%) (*Th* = 0.7)**
		
	**x** **(mm)**	**y** **(mm)**	**X direction** **(mm)**	**Y direction** **(mm)**		**X direction** **(mm)**	**Y direction** **(mm)**	
42	−100	−170	162	153	18.4	128	114	10.9
43	−130	−280	146	177	19.2	123	131	12.0
44	−90	−220	159	156	18.4	131	115	11.2
45	60	−160	168	147	18.4	124	113	10.4
46	0	−80	165	147	18.0	109	119	9.6
47	−10	−20	180	180	24.1	121	123	11.1
48	130	−100	149	154	17.1	119	123	10.9
49	150	−60	146	162	17.6	115	121	10.3
50	290	−40	187	170	23.6	119	132	11.7
51	320	20	198	195	28.7	146	130	14.1
52	110	200	161	157	18.8	128	113	10.8
53	130	60	222	203	33.5	190	186	26.3
54	−30	120	169	145	18.2	114	119	10.1
55	−80	350	157	176	20.5	104	117	9.0
56	−220	350	160	179	21.3	111	118	9.7
57	−350	0	281	207	43.3	212	178	28.1
58	−250	−60	165	164	20.1	123	141	12.9
59	−340	−80	212	159	25.1	161	138	16.5
60	−340	−80	212	159	25.1	161	138	16.5
61	−140	−290	168	186	23.2	127	128	12.1
62	−140	−290	168	186	23.2	127	128	12.1
63	200	−160	155	147	16.9	118	117	10.3
64	80	100	158	153	18.0	121	121	10.9
65	−220	−20	160	183	21.8	118	132	11.6
66	−150	−110	146	152	16.5	115	125	10.7
67	170	−140	151	151	17.0	116	120	10.4
68	170	−140	151	151	17.0	116	120	10.4
69	−180	−20	145	176	19.0	111	128	10.6
70	−140	−170	156	152	17.6	122	114	10.3
71	50	−330	196	213	31.0	131	152	14.8
72	110	350	182	182	24.6	134	119	11.9
73	−110	350	155	201	23.2	108	141	11.3
			**Accuracy rate 100%**		**Averaged percentage 21.8%**	**Accuracy rate 100%**		**Averaged percentage 12.5%**
